# Astrocytes actively support long-range molecular clock synchronization of segregated neuronal populations

**DOI:** 10.1038/s41598-023-31966-1

**Published:** 2023-03-24

**Authors:** Lidia Giantomasi, João F. Ribeiro, Olga Barca-Mayo, Mario Malerba, Ermanno Miele, Davide De Pietri Tonelli, Luca Berdondini

**Affiliations:** 1 Microtechnology for Neuroelectronics, Fondazione Istituto Italiano Di Tecnologia (IIT), 16163 Genova, Italy; 2 Neurobiology of miRNA, Fondazione Istituto Italiano Di Tecnologia (IIT), 16163 Genova, Italy; 3 Present Address: Circadian and Glial Biology Lab, Physiology Department, Molecular Medicine, and Chronic Diseases Research Centre (CiMUS), University of Santiago de Compostela, Santiago de Compostela, Spain; 4 Present Address: Centre de Nanosciences et de Nanotechnologies (C2N), CNRS UMR 9001, Université Paris-Saclay, 91120 Palaiseau, France; 5 Present Address: NanoPhotonics Centre, Cavendish Laboratory, Department of Physics, University of Cambridge, Cambridge, UK

**Keywords:** Cell biology, Neuroscience

## Abstract

In mammals, the suprachiasmatic nucleus of the hypothalamus is the master circadian pacemaker that synchronizes the clocks in the central nervous system and periphery, thus orchestrating rhythms throughout the body. However, little is known about how so many cellular clocks within and across brain circuits can be effectively synchronized. In this work, we investigated the implication of two possible pathways: (i) astrocytes-mediated synchronization and (ii) neuronal paracrine factors-mediated synchronization. By taking advantage of a lab-on-a-chip microfluidic device developed in our laboratory, here we report that both pathways are involved. We found the paracrine factors-mediated synchronization of molecular clocks is diffusion-limited and, in our device, effective only in case of a short distance between neuronal populations. Interestingly, interconnecting astrocytes define an active signaling channel that can synchronize molecular clocks of neuronal populations also at longer distances. At mechanism level, we found that astrocytes-mediated synchronization involves both GABA and glutamate, while neuronal paracrine factors-mediated synchronization occurs through GABA signaling. These findings identify a previously unknown role of astrocytes as active cells that might distribute long-range signals to synchronize the brain clocks, thus further strengthening the importance of reciprocal interactions between glial and neuronal cells in the context of circadian circuitry.

## Introduction

Most organisms have endogenous circadian clocks that coordinate physiological processes, including brain functions, and behavioral rhythms with respect to daily environmental cycles^1,2^. In mammals, such circadian system is organized in a hierarchy of multiple oscillators at molecular, cellular and organism level. At the molecular level, the circadian clock consists in a transcriptional/post-translational feedback loop (TTFL), in which the transcription factors Circadian Locomotor Output Cycles protein Kaput (CLOCK) and Brain and Muscle ARNT-like 1 (BMAL1, also known as ARNTL) drive the expression of *Per* and *Cry* genes, whose products lead to the inhibition of their own transcription^1^. This process oscillates with a 24 h period, producing the “ticking” of the biological clock. At the cellular level, multiple oscillating neurons are coupled to act as a single circadian unit leading to coordinated circadian signaling outputs^3^. Noteworthy, the discovery of astrocytes as clock cells^4,5^ and recently reported evidences on the role of astrocytes in entraining rhythmicity in neurons, in the circadian timekeeping and in behavior^5–11^, reveal a more complex cellular substrate of the circadian system than previously thought. At the organism level, the suprachiasmatic nucleus (SCN) of the hypothalamus is considered the master circadian pacemaker. The SCN is composed of a heterogeneous population of cells, including astrocytes and multiple neuropeptidergic classes of neurons^12^. The SCN receives direct inputs from the environment, which allow to synchronize to the day/night cycle. Among these inputs, light is the principal stimulus for external synchronization of circadian clocks, and in mammals this is mediated via the direct retinal innervation of the SCN derived from the retinohypothalamic tract (RHT)^12^. To orchestrate rhythms in the brain and throughout the body, the SCN is coupled with subsidiary oscillators. Such coupling mechanisms play therefore a key role in regulating the overall organism physiology and behavior^13^.

In the brain, various regions, including the prefrontal cortex, hippocampus, amygdala and dentate gyrus, exhibit circadian modulations in molecular expression. When isolated from the SCN in vivo, either by ablating the SCN or by encircling it with a knife cut, the periodicity in extra-SCN regions is abolished. This suggests that the central pacemaker within the SCN is responsible for driving near 24 h rhythmicity in other regions of the brain^14^. Interestingly, extra-SCN regions are characterized by a variation in phase and amplitude of rhythmic clock gene expression^15–17^. This reveals the presence of important regional differences in the temporal dynamics underlying local daily rhythms in the mammalian forebrain. Further, it underscores the complex temporal organization of subordinate circadian oscillators in the forebrain. However, considering that SCN neurons do not project to long distances in the brain^18^, the intercellular mechanisms that convey circadian cues from the master SCN clock to downstream extra-SCN brain regions remain unclear. Such communication among clocks could occur though neuronal connections, astrocytes connections or neuronal paracrine factors.

Here, we investigated two hypotheses for the circadian coupling mechanisms among neuronal populations. The first considers the role of paracrine factors released by neurons that may diffuse and synchronize distant neuronal populations. In particular, two main neurotransmitters are known to be involved in the regulation of the circadian clock: glutamate, which is involved in the photic entrainment of the circadian pacemaker^19^, and the γ-amino butyric acid (GABA), that was found to synchronize circadian firing rhythms in the dispersed SCN cell culture^20^ and to couple dorsal and ventral SCN circadian rhythms in acute SCN slices^21^. Despite different works suggest that GABA is likely involved in the entrainment and coupling of cellular circadian rhythms^20–22^, the mechanism of GABA action in the SCN is still a matter of debate. Controversy still exists on whether GABA acts as an excitatory or inhibitory neurotransmitter and on whether it acts as a synchronizer or destabilizer of the cellular rhythms^23–26^.

The second hypothesis considers the intercellular signalling among astrocytes as a potential pathway to entrain neuronal populations. This would be consistent with the recent accumulating evidence on the circadian role of astrocytes and the presence of a wide network of these cells in the brain^27^. Interestingly, both GABA and glutamate were also found to be involved in the astrocyte-mediated regulation of the circadian clock in the brain. Astrocyte clock was found to modulate circadian neuronal rhythms through GABA signaling^7^, and it was also found that astrocytes control circadian timekeeping in the SCN via glutamatergic signaling^5,8^.

As the disentangling of these mechanisms in vivo is cumbersome, we adopted a reductionist *lab-on-a-chip* approach based on a custom microfluidic device that allowed us to study these different signaling components in vitro. Indeed, this device allows to grow and compartmentalize distinct neural populations, connected or not through a network of astrocytes, to manipulate and quantify their clocks individually. At first, we investigated the entrainment of a distant and segregated neural population from a synchronized neural population. Then, we investigated the involvement of GABA and glutamate in the different inter-population signaling compartments, proposing a potential mechanism for the long-range entrainment of clock among neural populations.

## Results

### Astrocytes act as an active channel for neuronal clock synchronization

**Figure 1:**
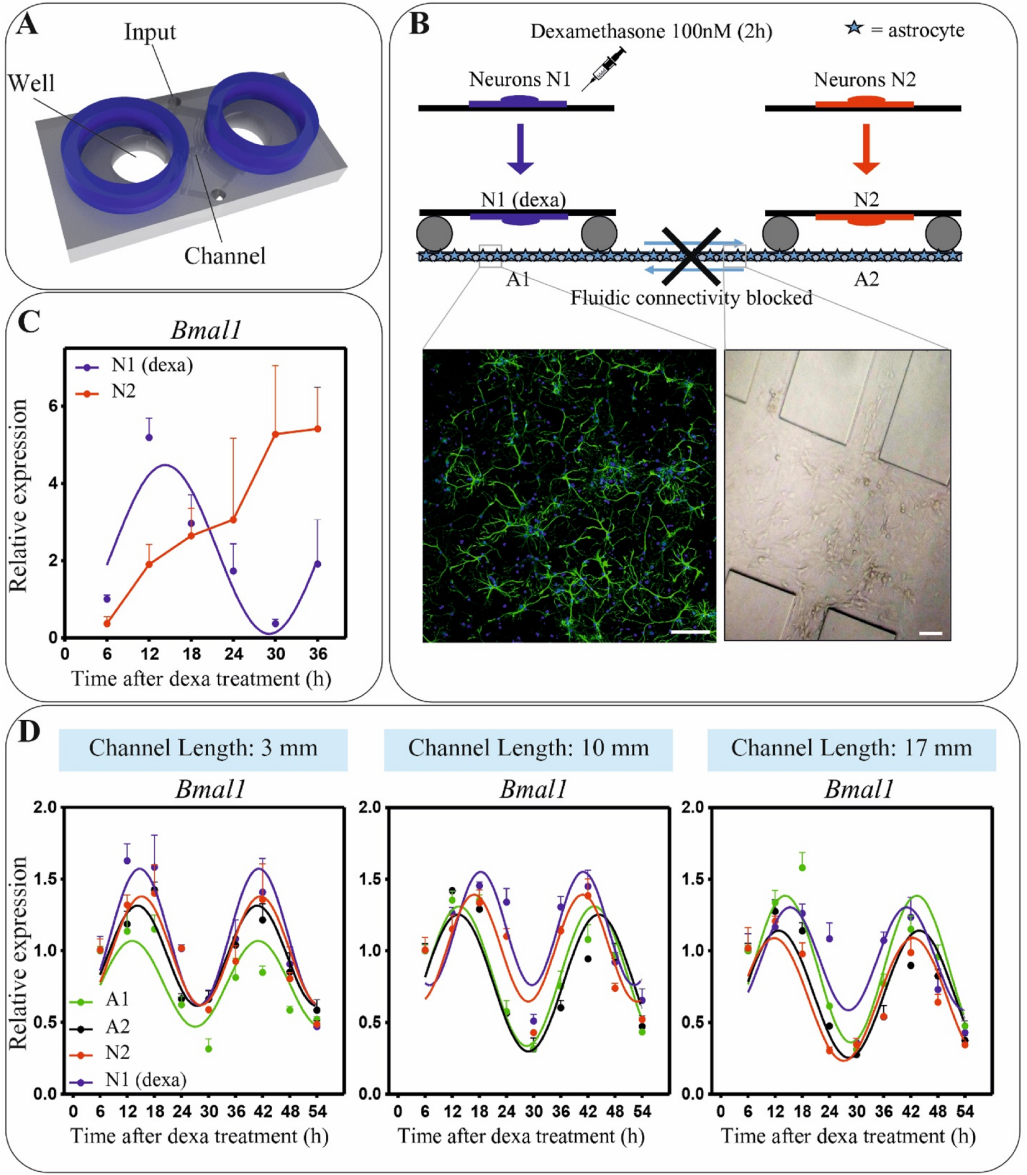
Astrocytes can mediate a long-range neuronal synchronization. (**A**) Illustration of the microfluidic device allowing to grow and compartmentalize distinct neural populations. (**B**) Schematic representation of the experimental protocol (upper panel); immunofluourescence image of astrocytes culture (GFAP, green; DAPI, blue) (lower panel, left); optical image of the center of the microfluidic channel with cultured astrocytes (lower panel, right). Scale bar: 50 µm. (**C**) *Bmal1* expression of N1 and N2 in a condition of fluidic connectivity blocked. N1 = neurons synchronized with Dexa 100 nM. N2 = asynchronous neurons. (**D**) *Bmal1* expression in all cellular populations in microfluidic devices with different lengths of the channel. Going from left to right, channel length is 3, 10 and 17 mm. A1, A2 = asynchronous astrocytes. In all graphs, *Bmal1* was analyzed at the indicate time points by qPCR and the mean ± s.e.m. of the cosine-fitted curves from an experiment performed in triplicate is represented.

To investigate whether astrocytes may act as a channel for the transmission of clock rhythms between two distant and segregated neuronal populations, we performed experiments on our *lab-on-a-chip* microfluidic device with two neuronal populations linked by a network of astrocytes. Such device was designed with two distant wells interconnected by a long microfluidic channel (80 μm high, 300 μm wide and, if not specified differently, 3 mm long). Six additional microfluidic channels, perpendicular to the interconnecting one, were used to continuously perfuse cell culture media in order to compartmentalize the two cell culture wells (Fig. 1A and Supplementary Fig. 1).

Cortical astrocytes were plated (or not, as control) in the microchannel and in the two wells of the microfluidic device, while neurons grown on a glass coverslip and synchronized with 100 nM of Dexamethasone (Dexa, 2 h) were used in one well (N1) and a population of asynchronous neurons in the other one (N2) (see Methods for details). Before starting the co-culture, the fluidic connectivity between the two chambers of the microfluidic device was blocked using the perfusion system. Under this condition, the only way of communication between the two populations of neurons is through astrocytes (Fig. 1B). Upon cell harvesting at different time points, the expression of the clock gene *Bmal1* was analyzed by real-time quantitative PCR (qPCR). Results show that in absence of astrocytes in the interconnecting channel the neuronal population N2 remains asynchronous (Fig. 1C, red curve), while N2 get synchronized in the presence of interconnecting astrocytes (Fig. 1D, left panel, red curve). The latter case, showing a rhythmic expression of *Bmal1* in the distinct neuronal populations, indicates that astrocytes can transfer circadian information among distant neuronal populations. Interestingly, by redesigning microfluidic devices with longer interconnecting channel, we found that astrocytes are able to transmit neuronal clock rhythms also at the longer (i.e. centimeter scale) distances and at least up to the tested condition of 17 mm (Fig. 1D; see Supplementary Fig. 2 for the analysis of the clock gene *Per2,* and Supplementary Fig. 3A for the circadian phase differences among astrocytes and neuronal populations). These results reveal the capacity of astrocytes to act as an active communication channel for a long-range neuronal synchronization.

In order to confirm that the observed N2 synchronization is due to a mechanism that starts from the synchronous neuronal population N1, we performed a series of control experiments. Firstly, we assessed whether the constant perfusion of cell culture media used to segregate the neuronal populations in the two wells might influence the expression profile of clock genes in astrocytes. In order to do this, a monolayer of asynchronous astrocytes (i.e. not treated with Dexa) was plated in the microfluidic device. Successively, astrocytes were harvested at different time points, before and during the perfusion, and the expression profile of the clock gene *Bmal1* was analyzed by qPCR (Supplementary Fig. 3B). Results show the presence of a rhythmic expression of *Bmal1* already before the perfusion. Most likely, this synchronization is induced by changes of the cell culture medium, an effect already reported by Prolo^4^ and by Barca-Mayo^9^. During perfusion, this rhythmicity is maintained with an increase of *Bmal1* expression, but without a significant increase in the amplitude of the oscillation or phase changes. The increase of *Bmal1* expression is likely due to the effect on astrocytes of B27, a culture supplement present in the neural culture medium that was used for the perfusion^28^.

Following these results, we performed the same experiment described above to reveal the capacity of astrocytes to synchronize neural populations, but with both neuronal populations (N1 and N2) asynchronous. In addition, in this case we changed the cell culture medium in astrocytes 24 h or 14 h before starting the co-culture with the neuronal populations (Supplementary Fig. 3C). Results show a circadian rhythmicity of *Bmal1* in astrocytes, whose phase changes according to the time the cell culture medium was changed. However, in both cases astrocytes are not able to synchronize any of the neuronal populations, thus confirming our result of an N1-to-N2 astrocytes mediated synchronization. Interestingly, this later result also reveals that, differently from the synchronization induced by a synchronous neuronal population, the synchronization of astrocytes induced by changes of the cell culture media is different and not effective to synchronize neurons.

### Neural paracrine factors show a limited spatial range in neuronal synchronization

**Figure 2:**
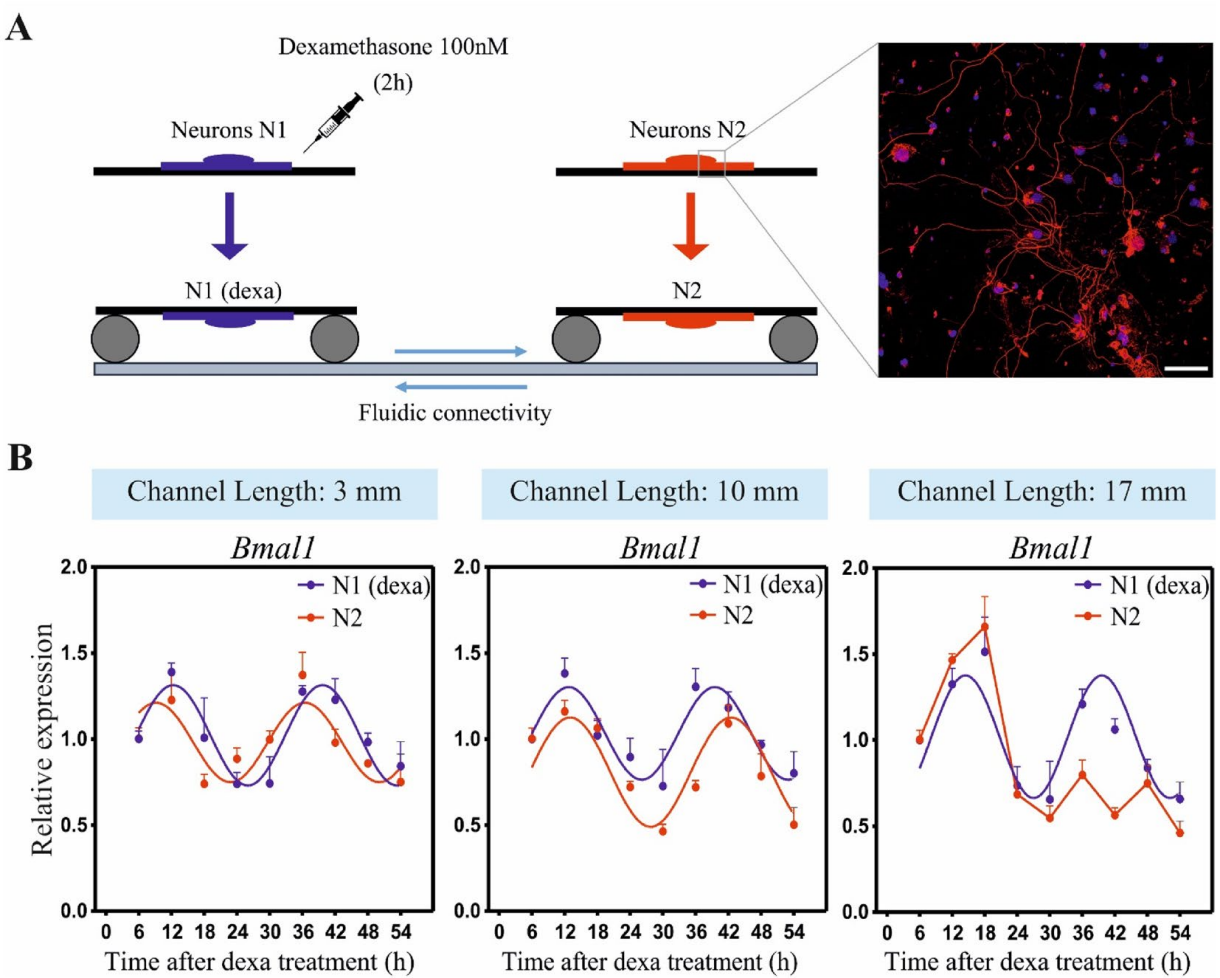
Neural paracrine factors can mediate a short-range neuronal synchronization. (**A**) Schematic representation of the experimental protocol (left); immunofluourescence image of neuronal culture (Neurofilament H, red; DAPI, blue) (right). Scale bar: 50 µm. (**B**) *Bmal1* expression in N1 and N2 in microfluidic devices with different lengths of the channel. Going from left to right, channel length is 3, 10 and 17 mm. N1 = neurons synchronized with Dexa 100 nM. N2 = asynchronous neurons. In all graphs, *Bmal1* was analyzed at the indicate time points by qPCR and the mean ± s.e.m. of the cosine-fitted curves from an experiment performed in triplicate is represented.

To investigate whether also paracrine factors released by synchronous neurons can synchronize a distant neuronal population, we performed the same experiment described above (Fig. 1B) but without astrocytes in the interconnecting channel and without perfusion. In this way, the fluidic connectivity between the two neuronal populations is allowed (Fig. 2A). Results show that synchronous neurons (N1) are able to synchronize an asynchronous neuronal population (N2) placed at a distance of 3 and 10 mm (Fig. 2B, see Supplementary Fig. 2 for the analysis of the clock gene *Per2*), confirming that there are paracrine factors released by N1 that can diffuse and synchronize N2. However, this ability is lost when the second neuronal population (N2) is placed at a distance ≥ of 17 mm. Of note, in this condition the asynchronous neural population seems receiving an input for the synchronization, but it is not entrained over time. This result confirms that neuronal paracrine factors can diffuse to synchronize neuronal populations, but it also reveals that their effect is limited to a shorter spatial range compared to astrocytes.

### GABA and glutamate are involved in the astrocytes-mediated synchronization

**Figure 3:**
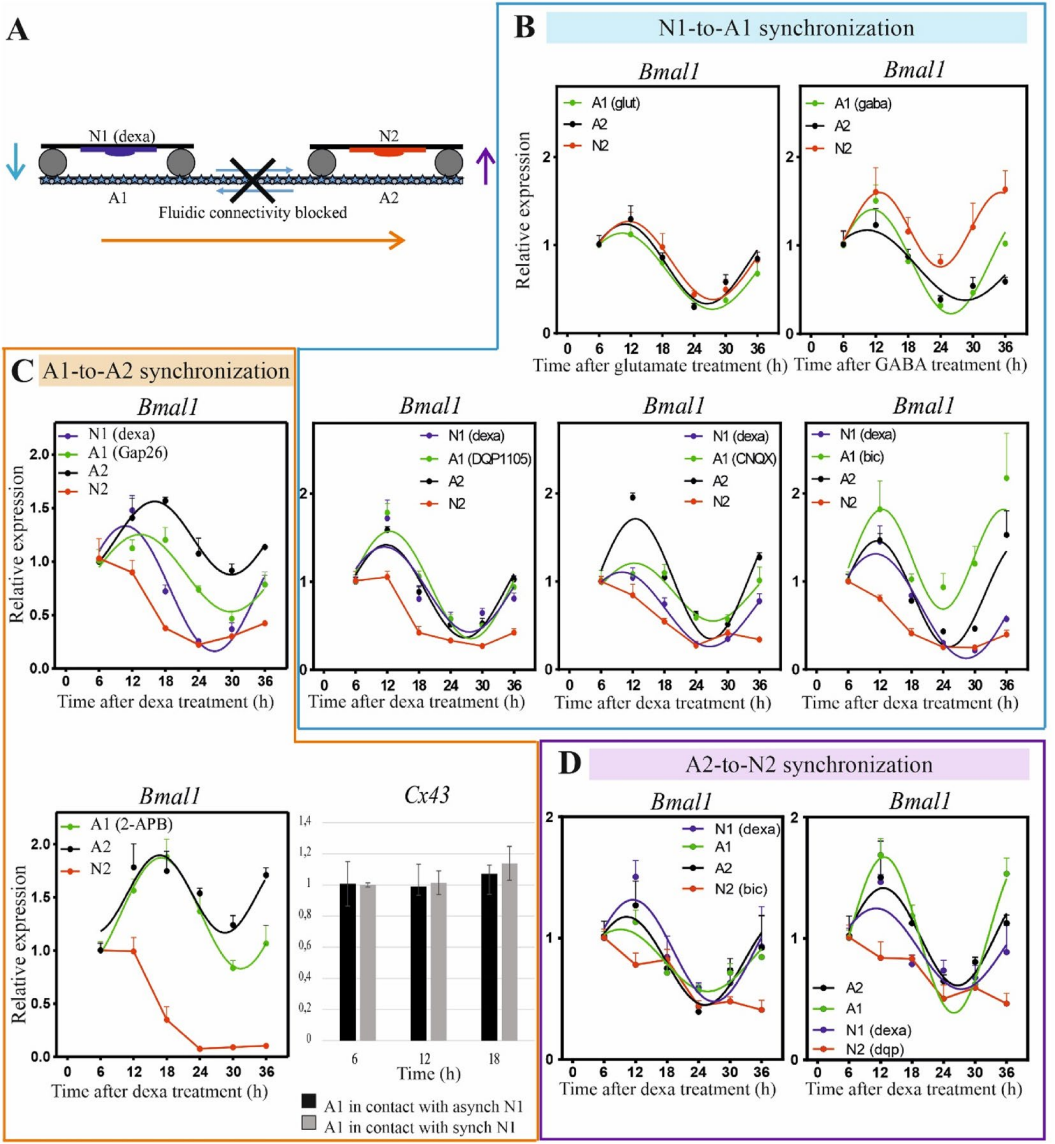
GABA and glutamate are involved in the astrocytes-mediated synchronization. (**A**) Schematic representation of the three different steps investigated for the mechanism involved in the astrocytes-mediated synchronization. (**B**) N1-to-A1 synchronization. Upper panels: *Bmal1* expression in A1, A2 and N2 after glutamate treatment (left panel) and GABA treatment (right panel). Lower panels: *Bmal1* expression in all cell populations after using GABA or glutamate receptors inhibitors in A1. From left to right, DQP1105 μM, CNQX 10 μM and Bicuculline 30 μM. (**C**) A1-to-A2 synchronization. Upper panel: *Bmal1* expression when intercellular communication between astrocytes is blocked with Gap26 100 μM in A1. Lower-left panel: *Bmal1* expression after blocking calcium signaling in A1 with 2-APB 100 μM. Lower-right panel: *Cx43* expression in astrocytes A1 in contact with asynchronous (black) or synchronous (grey) neurons. Paired t-test shows no significant difference between the two groups. (**D**) A2-to-N2 synchronization. *Bmal1* expression in the case of Bicuculline 30 μM (left) and DQP1105 50 μM (right) treatment in N2. Both *Bmal1* and *Cx43* were analyzed at the indicate time points by qPCR. In all graphs, the mean ± s.e.m. of the cosine-fitted curves from an experiment performed in triplicate is represented.

Having shown that astrocytes can act as a channel for the transmission of clock information among neuronal populations, next we investigated the mechanisms involved in such astrocytes-mediated synchronization. To do so, we distinguished and studied three major steps: (i) neurons (N1)-to-astrocytes (A1) synchronization; (ii) astrocytes (A1)-to-astrocytes (A2) synchronization; (iii) astrocytes (A2)-to-neurons (N2) synchronization (Fig. 3A).

#### Neurons (N1)-to-astrocytes (A1) synchronization

In the first step, astrocytes receive circadian information from the synchronous neuronal population. Most likely neurons release factors that, by acting on astrocytes, initiate a signaling that is transmitted through astrocytes and then determines the synchronization of other neurons. To investigate what are the possible factors involved in this neurons-to-astrocytes synchronization, we focused on the two main neurotransmitters glutamate and GABA.

Astrocytes were plated in the channel and the wells of the microfluidic device (A1–A2), co-cultured with asynchronous neurons placed in one well (N2) and treated with glutamate 400 μM (20 min) or GABA 100 μM (2 h) in the other well (A1). Both experiments were performed in a condition of fluidic connectivity blocked to ensure that the induced effects from one well to the other were mediated exclusively via the cell-to-cell communication of the astrocytic network. Note that neurotransmitters are delivered only to astrocytes (A1), and not directly to (N2) neurons, and that such neurotransmitters cannot reach the N2 neuronal population because of the blocked fluidic connectivity. As shown in Fig. 3B, neurons get synchronized in both cases, thus suggesting that both excitatory glutamate and inhibitory GABA actuate signalling in astrocytes that lead to a similar synchronization of the distant neuronal population N2. To confirm this result, three different additional experiments were performed by using inhibitors of glutamate and GABA receptors. Also in this case, the fluidic connectivity between the two chambers was blocked, astrocytes were placed in the channel and in the wells of the microfluidic device (A1–A2), a synchronous neural population was placed in one well (N1) and an asynchronous neural population in the other one (N2). To study the involvement of glutamate, A1 was incubated with the *N*-methyl-d-aspartate (NMDA) receptor antagonist DQP1105 (50 μM) or with the α-amino-3-hydroxy-5-methylisoxazole-4-propionic acid (AMPA)/kainite receptor antagonist CNQX (10 μM). Additionally, to study the involvement of GABA, A1 was incubated with the GABA_A_ receptor antagonist Bicuculline (30 μM). Results (Fig. 3B) show that N2 remains asynchronous regardless of the used inhibitor, thus confirming that both glutamate and GABA are required in the first step of the astrocytes-mediated synchronization observed between two segregated neuronal populations. It is interesting to note that astrocyte populations are synchronous for both conditions. To explain this, it should be reminded that, as stated in the results related to the expression of *Bmal1* in astrocytes before and during perfusion (Supplementary Fig. 3B), astrocytes are synchronous already before the perfusion due to changes of the cell culture medium. This means that the expression of *Bmal1* in astrocytes is always rhythmic, independently from the experimental conditions. However, our data suggest that such synchronization induced by changes of media differs from the one induced by a synchronous neuronal population and is not effective to synchronize neurons.

#### Astrocytes (A1)-to-astrocytes (A2) synchronization

To study the mechanisms involved in the second step (astrocytes-to-astrocytes synchronization), we first investigated whether the direct intercellular communication between astrocytes is required. To do so, we performed a synchronization assay under two different conditions: at first with segregated astrocytes populations, and secondly with astrocytes in direct contact.

For the first condition (Supplementary Fig. 4A, left panel), the A1 astrocyte population was plated on coverslips and grown until confluence, while the A2 population was plated on multiwell plates with paraffin feet that were successively used to avoid direct contact between the cultures^7^. In order to ensure that the synchronization of astrocytes is induced by a direct or indirect communication among cells and not by changes of the cell culture medium, before starting the co-culture experiment here we kept A2 in culture for some days without changing the cell culture medium. Astrocytes grown on coverslips (A1) were synchronized with 100 nM of Dexa (2 h) and placed upside-down in the dish containing the A2 astrocytes, thus sharing the same culture media. Then the A1 and A2 populations were harvested at different time points for subsequent analysis. For the second condition (Supplementary Fig. 4B, right panel), astrocytes were plated in the microfluidic channel and in the two wells of the microfluidic device. Once confluent, the fluidic connectivity between the two wells was blocked. Therefore, the only way of communication between the A1 and A2 populations was through astrocytes in the channel. Only one population (A1) was synchronized with 100 nM of Dexa (2 h). Finally, both A1 and A2 populations were harvested at different time points and the expression of clock genes analyzed.

Our experimental data (Supplementary Fig. 4B) show that synchronous astrocytes (A1) can induce rhythmic expression of *Bmal1* in initially asynchronous astrocytes (A2) only in the second condition, when the two populations are in direct contact. This result indicates that astrocytes require their direct cellular contact to synchronize their rhythms.

Next, we aimed at investigating this astrocyte-to-astrocyte intercellular signaling of clock rhythms. It is well known that gap junctions mediate intercellular communication among astrocytes by providing cytoplasmic continuity and that they are integral to formation of the functional syncytium that is required for the proper entrainment of circadian rhythms in the SCN^29–32^. For this reason, we blocked hemichannels and gap junctions between astrocytes in the first or in the second well of the microfluidic device by using Gap26 (100 μM), a selective inhibitor of Connexin-43 (Cx43). Interestingly, results (Fig. 3C upper panel, Supplementary Fig. 4C) show that, in both cases, the second neuronal population (N2) remains asynchronous and does not synchronize with the Dexa-treated N1 neuronal population. This result suggests that the intercellular communication between astrocytes through gap junctions is required for transmitting neuronal-clock rhythms among distant and segregated neuronal populations.

It has also to be noted that the increase of the astrocytic gap-junctional communication is not due to an upregulation of gap junction expression in astrocytes. For this, Cx43 expression was analyzed in A1 population of the two main experimental conditions, i.e. astrocytes in contact with synchronous neurons and astrocytes in contact with asynchronous neurons, and it does not show a significant difference (Fig. 3C, lower-right panel). This result is in line with other studies reported in literature where, in different experimental conditions, an increase of gap-junctional communication without an upregulation of Cx43 expression was shown^33,34^.

Another important aspect for the astrocyte-to-astrocyte communication is the intercellular calcium signaling (ICS). Inositol trisphosphate (IP3) appears to be the best candidate to play the role of “fuel” in the propagation of ICS in astrocytes^35^. Therefore, to investigate if calcium signaling is required for the transmission of clock rhythmicity among astrocytes, we performed a synchronization assay in the microfluidic device, by incubating one astrocyte population (A1) with 2-APB (100 μM, 2 h), an IP3 receptor antagonist, in a condition of fluidic connectivity blocked. Since 2-APB is not specific for astrocytes and blocks the release of calcium also in neurons, we did not use the N1 neuronal population, but synchronized directly A1 with Dexa 100 nM. Results (Fig. 3C, lower-left panel) show that the neuronal population (N2) remains asynchronous, thus suggesting that the intercellular calcium signaling in astrocytes is required for the synchronization of distant neuronal populations.

#### Astrocytes (A2)-to-neurons (N2) synchronization

For the third step (astrocytes-to-neurons synchronization), we started by taking into account what was reported in previous works. In 2017, we demonstrated that GABA, through GABA_A_ receptor signaling, mediates astrocyte to neuron communication^7^. In the same year, Brancaccio et al. showed that astrocytes release glutamate rhythmically and that blocking this release or uptake by dorsal SCN neurons suppressed and desynchronized circadian oscillations, thus suggesting a glutamatergic signaling between astrocytes and neurons^8^. Two years after, by two independent pharmacological approaches, i.e. interference with glutamate release by astrocytes (via Cx43 inhibition) and with neuronal glutamate sensing (via NMDA receptor antagonism), the same group demonstrated that glutamate is a necessary mediator of astrocytic control of circadian function in the SCN^5^.

By considering these findings, we investigated on our microfluidic device whether both GABA and glutamate are involved in the astrocytes-to-neurons synchronization. To do so, we blocked NMDA receptors or GABA_A_ receptors in the asynchronous neuronal population by adding DQP1105 50 μM or Bicuculline 30 μM respectively in the second chamber. As shown in Fig. 3D, the N2 neuronal population remains asynchronous, thus confirming the involvement of both GABA and glutamate in the astrocytes-to-neurons communication of clock rhythms.

### GABA is involved in the paracrine factors-mediated synchronization

**Figure 4:**
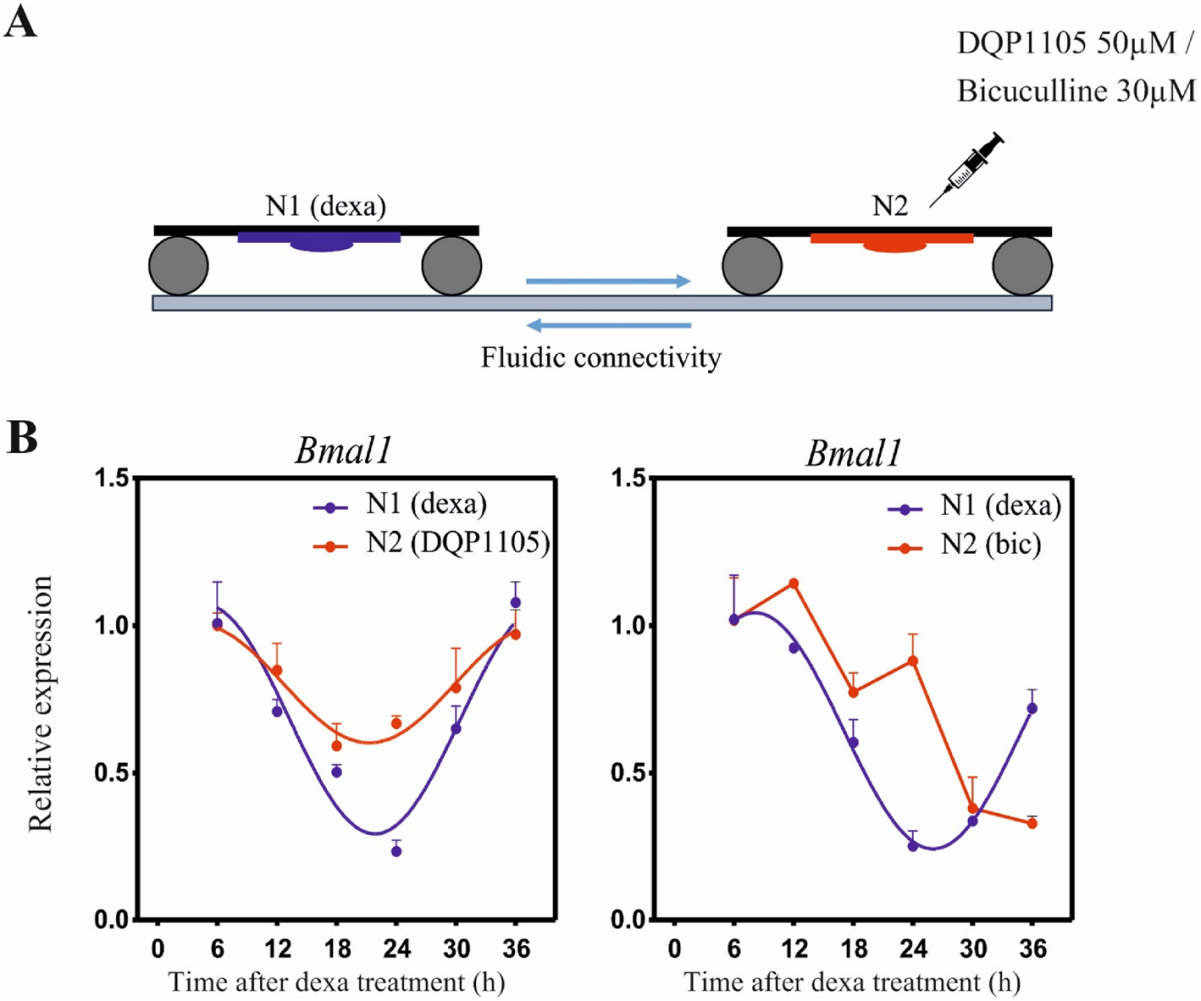
GABA is involved in the paracrine factors-mediated synchronization. (**A**) Schematic representation of the experimental protocol. (**B**) *Bmal1* expression in neurons in the case of DQP1105 50 μM (left) and Bicuculline 30 μM (right) treatment in N2. *Bmal1* was analyzed at the indicate time points by qPCR. All graphs show the mean ± s.e.m. of the cosine-fitted curves from an experiment performed in triplicate.

In Fig. 2, we showed that neural paracrine factors can entrain neuronal populations at short distances. In order to determine whether glutamate and GABA are involved, we performed two different experiments (Fig. 4A). By permitting a fluidic connectivity between the two wells, a synchronous neural population (N1) was placed in one well and an asynchronous neural population (N2) in the other one. In the first experiment, the N2 population was incubated with the NMDA receptor antagonist DQP1105 (50 μM); in the second, N2 was incubated with the GABA_A_ receptor antagonist Bicuculline (30 μM). Results (Fig. 4B) show that N2 synchronizes in the presence of the NMDA receptor antagonist, even if with a different amplitude, while it does not synchronize in the presence of the GABA_A_ receptor antagonist. This evidence suggests that for the paracrine factors-mediated synchronization of the two neuronal populations, only GABA is required. As related to glutamate, we postulate that, under this condition, it might have a role in sustaining the correct amplitude of the clock genes expression.

## Discussion

Circadian rhythms are essential in most organisms for coordinating the proper timing of physiology and behavior^36^. Although it is widely recognized that in mammalians the SCN of the hypothalamus is the master clock, a fascinating open question is how a few thousands of synchronous neurons can entrain billions of distant neuronal populations. Although communication among clocks can occur though neuronal connections, here we provide first evidence that astrocyte networks, by defining an active communication syncytium, can transmit circadian rhythms to entrain distant neuronal populations, possibly enabling the horizontal transfer of the rhythmic signaling among the circadian circuitries. Interestingly, such astrocyte-mediated entrainment expands the spatial reach compared to diffusive paracrine factors.

In our experiments we developed a *lab-on-chip* device that allowed us to implement a reductionist in vitro model system for exquisitely disentangling differences and evaluating signaling mechanisms involved in the entrainment of two neural populations when mediated either by the release of paracrine factors or by an astrocytic interconnection. By taking advantage of this model system, we demonstrate that the activity of astrocytes can synchronize the clock of two neuronal populations, at least up to a distance of 17 mm. Differently, in absence of astrocytes, paracrine factors released from a synchronous neuronal population can synchronize an asynchronous neuronal population at shorter range and up to a distance of 10 mm (considering the measured distances of 3, 10 and 17 mm).

In this model system, when the fluidic connection between neural populations is allowed, and astrocytes are not present in the interconnecting microchannel, paracrine signaling among the two cell populations is governed by diffusion of small amounts of neural factors through the microchannel. Given the long circadian period, this process occurs under very slow frequency variations. Under these conditions of quasi equilibrium, the different effects observed at steady state for paracrine factors in devices of 10 mm and 17 mm in channel length can be explained by the difference in dilution given by the different volume of the two devices, i.e. 0.168 µl. Differently, at the beginning of our experiments the synchronized neural population placed in the first well introduces a release of factors that change their initial concentration in the cell culture media. This is sufficient to induce a clock gene expression response as observed in our experiments with devices having 17 mm in channel length. Interestingly, this result also suggests that continuous variations of paracrine factors (at sufficient amplitude) are required to entrain the second neuronal population. It has to be noted that all these experiments were performed with a fine control on the volumes of media and in triplicate. These data, however, cannot be directly compared with an in vivo situation, and should be taken as a figure of merit to value the spatial reach potential of the astrocytic-mediated entrainment with respect to the entrainment of neurons mediated by the diffusion of paracrine factors.

**Figure 5:**
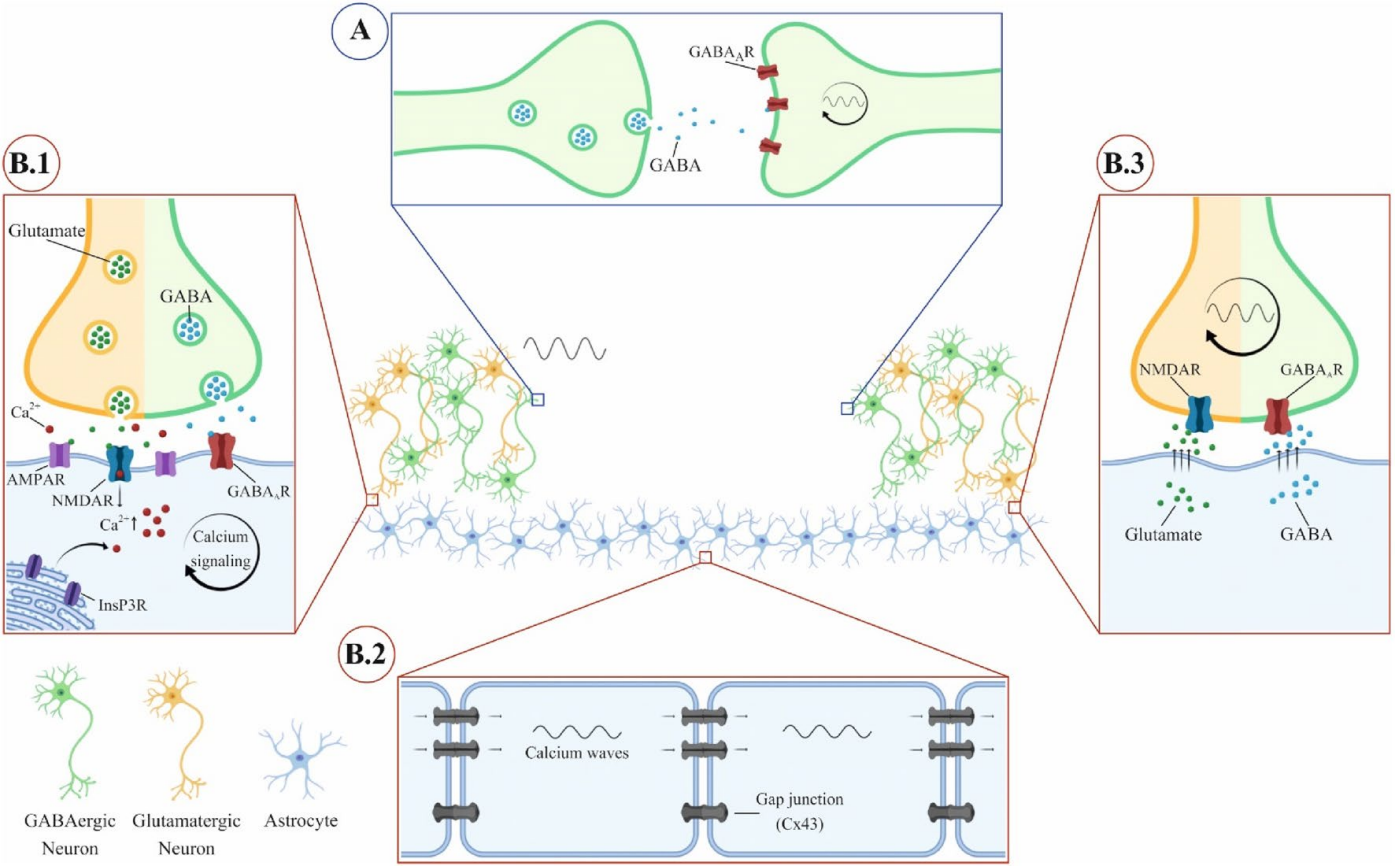
Mechanisms proposed for the synchronization of distant neuronal population. Two pathways have been identified in this work. (**A**) Paracrine factors-mediated synchronization. Neurons release GABA that diffuses and, by binding GABA_A_ receptors, triggers a signaling that allows the synchronization of neurons. (**B**) Astrocytes-mediated synchronization. Neurons release GABA and glutamate, which bind respectively GABA_A_ receptors and AMPA or NMDA receptors present on astrocytes. This binding determines an increase in the intracellular concentration of Ca^2+^, triggering a calcium signaling (**B.1**). Through GAP junctions, calcium waves propagate in the astrocyte network (**B.2**). This signaling ends with the release of glutamate and GABA from astrocytes that, binding respectively NMDA and GABA_A_ receptors on neurons, trigger the synchronization of neurons (**B.3**). Created with Biorender (biorender.com).

On the other hand, our results suggest that both paracrine- and astrocytic-mediated entrainment of neural populations might occur in vivo, and parallel the neural connections mediated entrainment. As illustrated in Fig. 5, these parallel pathways (A and B in the figure) might act concomitantly to ensure a robust synchronization of cellular clocks in the whole brain. Indeed, while synchronous neurons release paracrine factors that can locally diffuse to synchronize nearby neurons and astrocytes, the so entrained astrocytes can actively propagate this synchronization signal to entrain more distant neurons that can restart the same process.

Our experiments based on pharmacological manipulations in the three compartments of our model system (i.e. neurons-to-astrocytes synchronization, astrocytes-to-astrocytes synchronization, and astrocytes-to-neurons synchronization), reveal that the neuronal population synchronized by Dexa entrains astrocytes in the first compartment through the release of GABA and glutamate binding astrocytic GABA_A_ receptors and AMPA or NMDA receptors, respectively. In astrocytes, this binding determines an increase in the intracellular concentration of Ca^2+^, which triggers a calcium signaling, and calcium waves propagate in the astrocyte network through astrocytic GAP junctions. This signaling ultimately leads to the release of glutamate and GABA from astrocytes in the second compartment that trigger the synchronization of the distant neural population by binding respectively NMDA and GABA_A_ receptors on neurons. This is in line with the findings reported by Brancaccio^5,8^ and Barca-Mayo^7^, in which, by using different approaches, it was shown that astrocytes can synchronize neurons respectively through a glutamate and GABA signaling.

As reported in literature, GABA binding to its astrocytic receptor activates astrocytes, and leads to glial calcium transients, which in turn can induce the release of gliotransmitters, rendering GABA an important mediator of neuron-glia interactions^37–39^. The same behavior was reported for glutamate, which in astrocytes induces an intracellular calcium increase through the activation of NMDA and AMPA receptors^40,41^, induces calcium waves for a long-range signaling^42,43^ and upregulates gap-junctional communication^34^. In addition, also the NMDA receptor in astrocytes was reported to exert a non-canonical metabotropic-like function, regulating Ca^2+^ exit from the endoplasmic reticulum and consequently increasing the intracellular calcium^44^. Interestingly, the extent of the propagation of intercellular waves in astrocytes depends on the glutamate concentration: at low concentrations (< 1 µM), distinct areas of an astrocyte flickered asynchronously, and intracellular waves typically propagated only through portions of cells. At higher concentrations (1 to 10 µM), Ca^2+^i waves more commonly propagated through entire cells, and intracellular waves began to propagate into neighboring cells. At still higher concentrations (10 to 100 µM), intercellular waves began to propagate over long distances^42^. This could explain why only astrocytes in contact with synchronous neurons are able to synchronize a distant neuronal population. Indeed, differently from asynchronous neurons, synchronous neurons release factors in a circadian manner. Consequently, only at certain period of time the glutamate concentration is high enough to trigger the formation of calcium waves is astrocytes. This explanation would be in line also with the study of Ananthasubramaniam et al., showing that timing of coupling determines synchrony and entrainment in the mammalian circadian clock^45^.

We have also observed that blockade of the spreading of calcium waves among astrocytes by using either an IP3 receptor antagonist or a gap junctions blocker specific for astrocytes, leaves the distant neuronal population asynchronous. This suggests that astrocyte gap junctions, and consequently the astroglial intercellular communication, and Ca^2+^ signaling are required for the transmission of the molecular clock synchronization to neurons. Of course, these findings do not exclude the involvement of ATP as another messenger for the spreading of calcium waves among astrocytes^46,47^ and further analysis are needed to better understand the mechanisms involved in the transmission of molecular clock through astrocyte networks.

Although with this work we cannot state that the astrocytic molecular clock is involved in the neuronal synchronization, we still can conclude that our study, performed with cortical neurons, reveals the active functional role of astrocytes activity in the long-range transmission of circadian information among neural populations. Furthermore, given the need of astrocytes of both glutamate and GABA for the synchronization of distant neuronal populations, our findings might also have implications on the synchronization among the SCN and distant neural clocks.

By revealing that astrocytes can act as an active channel for the synchronization of distant neural clocks, this work strength the role of astrocytes in the circadian field.

## Methods

### Ethical statement

Experiments presented herein were performed at the Italian Institute of Technology (IIT). All animal procedures carried out in this work were approved by the institutional IIT Ethics Committee and by the Italian Ministry of Health and Animal Care (Authorization No. 110/2014-PR of the 19th of December 2014). All methods were carried out in accordance with relevant guidelines and regulations, and are reported in accordance with ARRIVE guidelines.

### Microfluidic device design and fabrication

The realization of the microfluidic device consists in mounting on a 4’’ glass wafer a micro-structured polydimethylsiloxane (PDMS) (Sylgard 184, Sigma-Aldrich) layer. This layer defines the microfluidic circuitry and it was obtained as replicas from a Si master by using the so-called micromolding technique (Supplementary Fig. 1A–C). Positive structures on the Si master were obtained by patterning a Cr layer with optical lithography and dry etching the Si substrate using DRIE (Deep Reactive Ion Etching). To do so, 4″ p-type Si wafers were first cleaned by subsequent acetone, isopropyl-alcohol and deionized water (DI) washing. Next, positive tone photoresist (MEGAPOSIT SPR 200, MicroChem) was deposited by spin coating (4000 rpm) onto the Si wafer and baked at 115 °C for 2 min. The microfluidic circuitry was patterned by exposing the photoresist to UV light (MA-6, SUSS MicroTec mask align) through a laser written lithography mask and developed for 1 min in Microposit MF-319 developer. Successively, a 200 nm thick Cr layer was deposited by e-beam evaporation (Kenosistec KE500ET) at 1.5 Å/s deposition rate and unwanted Cr remaining on the photoresist was lifted-off in acetone (overnight). The Cr patterned layer was then used as mask for dry-etching process. A DRIE Bosh process (SENTECH SI500, ICP-RIE) was employed to etch the Si for 80 μm and the depth measured by mechanical profilometer (Dektak 150). This thickness defines the final depth of the microfluidic channel between the two wells in the PDMS replicas.

The processed Si wafer was then cleaned by O_2_ plasma (100 W, 300 s) and the Cr layer removed in a Cr etchant solution (Chrome Etch 18, Micro Resist Technology GmbH). Finally, a fluorosilane anti-sticking layer was deposited onto the Si wafer to facilitate PDMS removal in the molding process. To do so, a 250 μl of Perfluorooctyltriethoxysilane (POTS, Alfa Aesar L16606) were dispensed onto a glass slide and placed under vacuum with the Si wafer for 1 h. The wafer was then baked on a hot plate at 80 °C for 5 min.

PDMS was prepared by mixing the curing agent and PDMS monomers in a ratio 1:10. After degassing under vacuum, it was deposited onto the structured Si mold (approximately 4 mm in height) and cured at 65 °C in oven for 2 h. Then, the cured PDMS was peeled-off from the Si mold, thus obtaining negative microfluidic structures, i.e. replica of the positive structures defined onto the Si wafer. A hole punch of 8 mm in diameter was used to realize the two wells through the PDMS layer, while a 1 mm in diameter hole punch was used to make the holes for the input microfluidics used to compartmentalize the cultures. Next, this PDMS structured layer was mounted on a glass wafer, previously cleaned by subsequent acetone, isopropyl-alcohol and DI water washing. To allow the fixing of the PDMS on the glass wafer, the surfaces of both the substrate and the PDMS were treated in O_2_ plasma (20 W, 30 s). Finally, glass cylinders (15 mm in diameter, 10 mm in height) were fixed on the PDMS device, by gluing them with more PDMS, to create the cell culture wells. As PDMS is hydrophobic, it is necessary to make it hydrophilic to allow cell growth. To do so, the device was treated in O_2_ plasma (100 W, 120 s).

The realized PDMS microfluidic devices consist of two chambers communicating through a 3, 10 or 17 mm long microfluidic channel (80 μm high, 300 μm wide). Additional microfluidic channels, perpendicular to the interconnecting one, are used to continuously perfuse media in order to compartmentalize in a fluidic manner the two cell culture wells.

### Microfluidic device validation

Microfluidic testing of the functionality of the vertical fluidics developed to compartmentalize the two cell culture wells was performed by applying a Coomassie Brilliant Blue dye (Sigma 27,815) in one of the wells, both with/without vertical perfusion (Supplementary Fig. 1D and Supplementary Video). Such perfusion was performed using Milli-Q water. To check there was no unwanted diffusion through the channel when perfusion (i.e. a positive pressure) was applied, the segregation of the dye was monitored for 54 h.

### Primary astrocyte culture

Primary monolayer cultures of astrocytes were established from cerebral cortices of neonatal (P1–P3) Sprague–Dawley rats and maintained at 37 °C in a humidified atmosphere of 5% CO_2_. The following solutions and media were used: Hanks Balanced Salt Solution (HBSS) (Sigma H6648); digestion solution—Dispase II 2 mg/ml (Roche 04942078001) in Phosphate-Buffered Saline (PBS) (Thermo Fisher Scientific 10010056) + DNAse I 25 µg/ml (Sigma D5025) in PBS; complete medium—DMEM/F-12 (Sigma D6421) supplemented with 1% Glutamax (Thermo Fisher Scientific 35050038), 1% Penicillin/Streptomycin (Sigma P4333) and 10% FBS (Sigma F7524). Briefly, pups were removed and decapitated, and the brains were extracted from the skulls and placed in cold HBSS. After dissection, cortices were disaggregated by pipetting, placed in the digestion solution and incubate in water bath at 37 °C for 30 min. Cell solution was centrifuged at 900 rpm for 5 min, and the supernatant was removed. The cell pellet was resuspended in complete medium (considering 10 ml per pup). The solution was filtered with a cell strainer (Biologix 15-1040, 40 µm pore size), and cells were plated in flasks (considering 1 flask per pup). The day after plating, the medium was changed to remove dead cells. The cultures were maintained at 37 °C in a humidified atmosphere of 5% CO_2_ for 1 week and thereafter cells were trypsinized and subcultured for the different experiments.

### Primary neuronal culture

Primary neuronal cultures were established from cerebral cortices of embryonic day 18 (E18) Sprague–Dawley rats and maintained at 37 °C in a humidified atmosphere of 5% CO_2_. The following solutions and media were used: Hanks Balanced Salt Solution (HBSS) (Sigma H6648); digestion solution—Trypsin 0,125% (Thermo Fisher Scientific 25050014) in HBSS + DNAse 0,25 mg/ml (Sigma D5025) in HBSS 5 mM CaCl_2_; complete Neurobasal-Neurobasal medium (Thermo Fisher Scientific 21103049) supplemented with 2% B27 (Thermo Fisher Scientific 17504044), 1% Glutamax (Thermo Fisher Scientific 35050038) and 1% Penicillin/Streptomycin (Sigma P4333); FBS (Sigma F7524).

Briefly, embryos were removed and decapitated, and the brains were extracted from the skulls and placed in cold HBSS. After dissection, cortices were placed in the digestion solution and incubate in water bath at 37 °C for 30 min. Few ml of complete Neurobasal + 10% FBS were added to the cell solution. It was centrifuged at 1200 rpm for 5 min, and the supernatant was removed. The cell pellet was resuspended in fresh complete Neurobasal + 10% FBS and gently pipetted for not more than 10 times with P1000 pipette. The solution was filtered with a cell strainer (Biologix 15-1040, 40 µm pore size), centrifuged at 700 rpm for 7 min, and the supernatant was removed. The cell pellet was resuspended in complete Neurobasal. Cell viability at the time of isolation was determined by a Trypan Blue Exclusion Assay (Sigma T8154). Cells were plated at a density of 90.000 cells/well onto coverslips coated with poly-D-lysine 0.1 mg/ml (Sigma P6407) in 24-well dishes. Five days after plating, half of the medium was added, and subsequently every 4–5 days half of the medium was changed. Neuronal cultures were maintained for up to 3 weeks in vitro before being used for the different experiments.

### Microfluidic device experiments and treatments

In experiments, the microfluidic device was used either with astrocytes interconnecting the two wells in which neuronal populations are placed, or without astrocytes to study the effect of released paracrine factors.

In the first experimental condition, cortical astrocytes were plated in the microchannel, through the same holes used for the perfusion, and in the two wells of the microfluidic device. Once confluent (3–4 DIVs) the medium in the device was replaced with 50% complete Neurobasal + 50% conditioned Neurobasal. The day after, the fluidic connectivity between the two chambers of the microfluidic device with cultured astrocytes was blocked by perfusing 50% complete Neurobasal + 50% conditioned Neurobasal. Then, neurons at 22–24 DIVs, that were grown separately on coverslips, were synchronized with Dexamethasone 100 nM (Sigma D4902) for 2 h and successively placed upside-down in one well of the device (N1). An untreated asynchronous neuronal culture (N2) grown in the same conditions of N1 was placed in the other well of the device. In the second experimental condition, i.e. without astrocytes, experiments were performed by placing neuronal cultures grown on coverslips in the two wells.

All cellular populations in the two wells (i.e. N1, A1 and N2, A2) were harvested at different time points for subsequent qPCR analysis, and all experiments were performed in triplicate by exploiting the integration of multiple distinct microfluidic devices on the same PDMS layer. For the different experimental conditions detailed in the results section, the following compounds were used: DQP1105 50 µM (Tocris Bioscience 380560-89-4), Bicuculline 30 µM (Sigma B7561), Glutamate 400 µM (Sigma 49,449), GABA 100 µM (Sigma A2129), CNQX 10 µM (Tocris Bioscience 479347-85-8), Gap26 100 µM (AnaSpec AS-62644), 2-APB 100 µM (Tocris Bioscience 524-95-8).

### RNA isolation and quantitative real-time PCR (qPCR)

Cells were harvested at the appropriate time points and at a time interval of 6 h. Total RNA was extracted using TRIzol reagent (Invitrogen 15596018) following the manufacturer’s instructions. RNA was further cleaned using a DNase I Kit (Sigma AMPD1). Complementary DNA (cDNA) was obtained by reverse transcription of 0.3 mg of total mRNA using the M-MuLV-RH First Strand cDNA Synthesis Kit (Experteam R01-500) following the manufacturer’s instructions. Real-time reverse transcriptase–PCR was done using the 7900HT Fast Real-Time PCR System (Applied Biosystems). For a 10 ml reaction, 9 ng of cDNA template was mixed with the primers (final concentration: 400 nM each primer) and with 5 µl of 2 × iTaq Universal SYBR Green Supermix (Biorad 172–5124). The reactions were done in duplicates using the following conditions: 30 s at 95 °C followed by 40 cycles of 15 s at 95 °C and 60 s at 60 °C. The primers used are listed in Table 1. Gapdh transcript was used as control.

**Table 1:** Primers used in this work for qPCR (Gapdh transcript as control).

PRIMER	SEQUENCE 5′-3′
Gapdh—Forward	TGTGTCCGTCGTGGATCTGA
Gapdh—Reverse	CCTGCTTCACCACCTTCTTGA
Bmal1—Forward	CCGATGACGAACTGAAACACCT
Bmal1—Reverse	TGCAGTGTCCGAGGAAGATAGC
Cx43—Forward	ACAGCTGTTGAGTCAGCTTG
Cx43—Reverse	GAGAGATGGGGAAGGACTTGT

### Immunofluorescence analysis

Astrocytes and neurons grown on coverslips were fixed in PFA 4% for 15 min and washed in PBS before being processed for immunostaining. Fixed cells were permeabilized with 0.1% Triton X-100 (Sigma T9284) in PBS (PBST) for 20 min at room temperature (RT), blocked with normal goat serum (NGS, 10%, Sigma G9023) in PBST for 1 h at RT, and subsequentely incubated at 4 °C overnight with the primary antibody rabbit anti-glial fibrillary acidic protein (GFAP, DAKO Z0334, 1:250) and chicken anti-neurofilament H (Millipore AB5539, 1:1000) diluted in PBST + 5% NGS. The following day, cells were washed three times with PBST and incubated for 45 min at RT with Alexa-488, -647 secondary antibodies (Invitrogen) diluted 1:1000 in PBST + 5% NGS. Nuclei were counterstained with DAPI. Cells were then washed three times with PBST and once with PBS, mounted and imaged using a Leica SP5 inverted confocal microscope with 40 × objective lenses (Leica Microsystems).

### Statistical analysis

Data are presented as mean ± s.e.m. and were analyzed and graphed using Prism 5 (GraphPad, San Jose, CA, USA). As in other studies, the circadian period was determined using a nonlinear regression curve fitting of the measured time series of levels of expression using a single-component cosinor model^48^, described by the following equation:

$$Y\left(t\right)=MESOR+Acos(f*t+\Phi )$$

where *Y(t)* is the level of expression for a given time point *t*, *MESOR* is the Midline Statistic Of Rhythm (rhythm-adjusted mean), *A* is the amplitude of the level of expression, *f* is the frequency of the oscillation, and *Φ* is the acrophase. This was performed in GraphPad Prism using the “XY analyses – Nonlinear Regression (curve fit)” (Baseline = 1, rule: *YMID, Amplitude A = 1, rule: *(YMAX-YMIN), Frecuency f = 0.2618, rule: (initial value, to be fit), acrophase Φ = 1.0, rule: *(Value of X at YMAX); no default constraints set for these parameters). The quantified frequency (f) was used to determine the circadian period T with the following equation: T = 2π/f. We considered synchronous a population in which *Bmal1* has a rhythmicity whose period is 24 ± 6 h. When such parameter is satisfied, we used cos fitting; otherwise, we just used a line connecting all the time-points. For comparison between groups, a paired t-test was used: *P* < 0.05 was considered as statistically significant and the significance is marked by *P < 0.05; **P < 0.01 and ***P < 0.001.

## Supplementary Information

Supplementary Information 1.

Supplementary Video 1.

## Data Availability

All data generated and analyzed during the current study are available from the corresponding author on reasonable request.
