# Supplementary material for: Astrocytes actively support long-range molecular clock synchronization of segregated neuronal populations
Source: Sci Rep. 2023 Mar 24;13:4815. doi: 10.1038/s41598-023-31966-1 (PMC10038999; doi:10.1038/s41598-023-31966-1)
Supplement: Supplementary file 1 — Supplementary Information 1. [file 41598_2023_31966_MOESM1_ESM.pdf]

Supplementary Figures

Astrocytes actively support long-range molecular clock synchronization of segregated neuronal populations

Lidia Giantomasi[1], João F. Ribeiro[1], Olga Barca-Mayo[2,3], Mario Malerba[1,4], Ermanno Miele[1,5], Davide De Pietri Tonelli[2], Luca Berdondini[1,*]

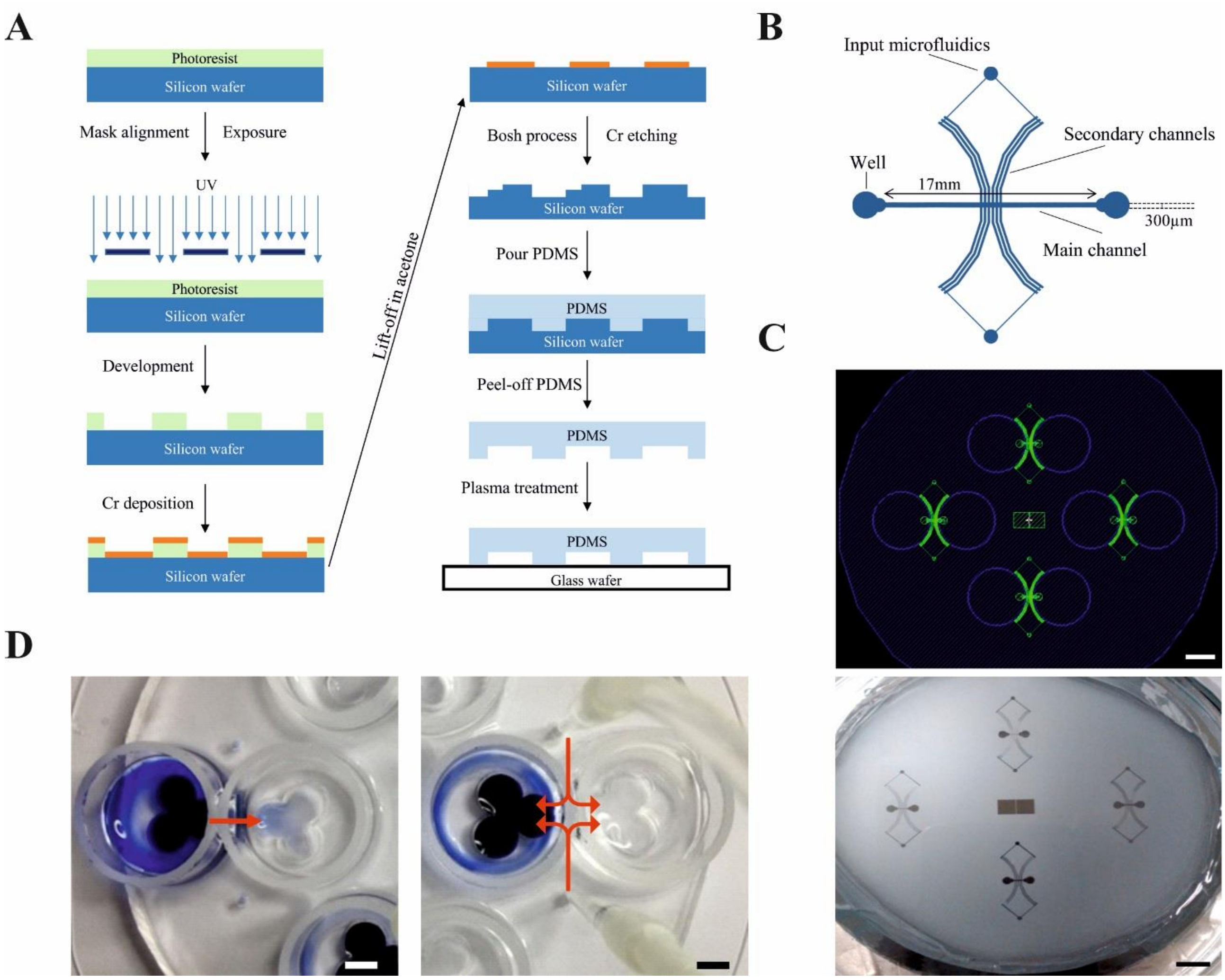

Supplementary Figure 1: Microfluidic device fabrication and validation test. A) Schematic illustration of the fabrication process of the PDMS microfluidic device. B) Illustration of the microfluidic circuitry. C) Layout of the microfluidic circuitry (upper panel), view of a Si mold (lower panel). Scale bar: 0.5 cm. D) Compartmentalization testing by using a Coomassie Brilliant Blue dye. Case without vertical perfusion (left panel): as shown the blue dye in the left well can flow in the right well. Case with vertical perfusion (right panel): as shown the blue dye remains confined in the left well. Scale bar: 0.5 cm.

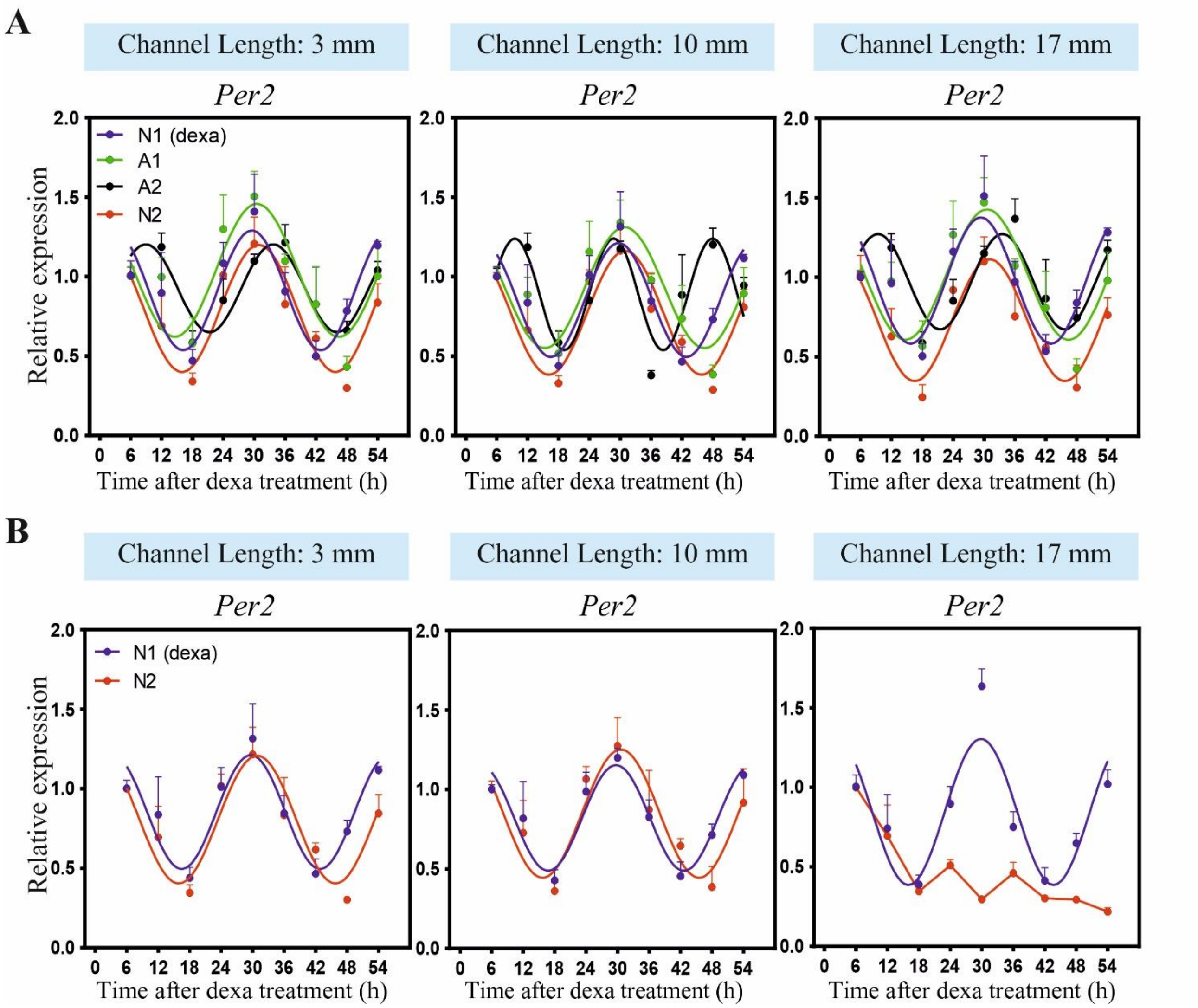

Supplementary Figure 2: *Per2* expression with or without the interconnecting astrocytic network. A) *Per2* expression in all cellular populations in presence of an interconnecting astrocytic network in microfluidic devices with different lengths of the channel. Going from left to right, channel length is 3, 10 and 17 mm. N1 = neurons synchronized with Dexa 100 nM. N2 = asynchronous neurons. A1, A2 = asynchronous astrocytes. B) *Per2* expression in all neuronal populations in absence of an interconnecting astrocytic network in microfluidic devices with different lengths of the channel. Going from left to right, channel length is 3, 10 and 17 mm. N1 = neurons synchronized with Dexa 100 nM. N2 = asynchronous neurons. In all graphs, *Per2* was analyzed at the indicate time points by qPCR and the mean ± s.e.m. of the cosine-fitted curves from an experiment performed in triplicate is represented.

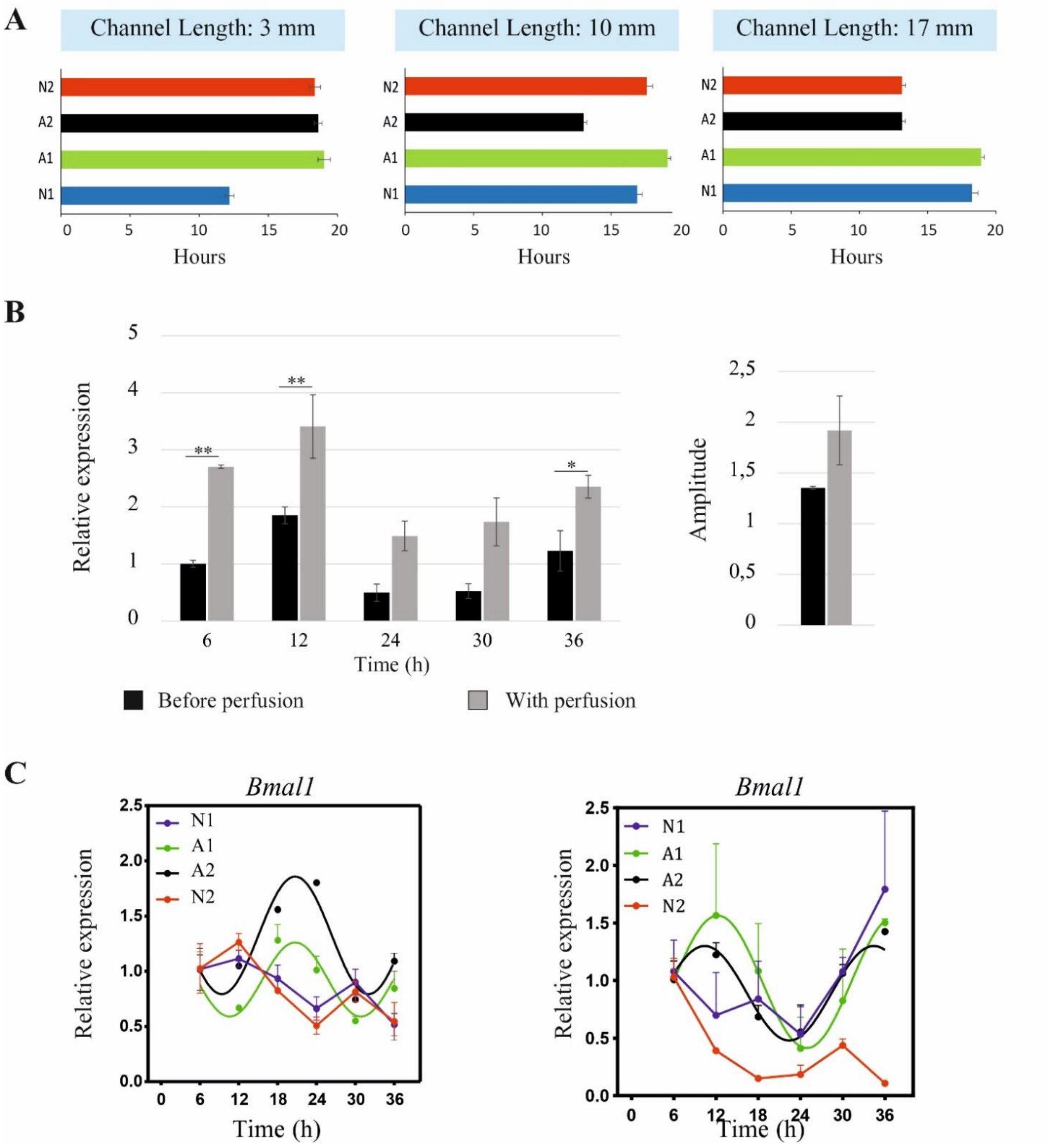

Supplementary Figure 3: Effect of perfusion and medium changing on astrocytic *Bmal1* expression. A) Circadian phase analysis in all cellular populations. Going from left to right, channel length is 3, 10 and 17 mm. N1 = neurons synchronized with Dexa 100 nM. N2 = asynchronous neurons. A1, A2 = asynchronous astrocytes. B) Left: *Bmal1* expression in astrocytes before (black) and with (grey) perfusion used to segregate the wells of the microfluidic device. *Bmal1* was analyzed at the indicate time points by qPCR. The graph shows the mean ± s.e.m. from an experiment performed in triplicate. Right: Amplitude of *Bmal1* oscillation in astrocytes before (black) and with (grey) perfusion. Two-way ANOVA: *P<0.05, **P<0.01 and ***P<0.001 versus astrocytes before perfusion. C) *Bmal1* expression in each cellular population (N1, A1, A2, N2), with medium changed 14 h (left panel) end 24 h (right panel) before starting the experiment. N1, N2 = asynchronous neurons. A1, A2 = asynchronous astrocytes. *Bmal1* was analyzed at the indicate time points by qPCR. Both graphs show the mean ± s.e.m. of the cosine-fitted curves from an experiment performed in triplicate.

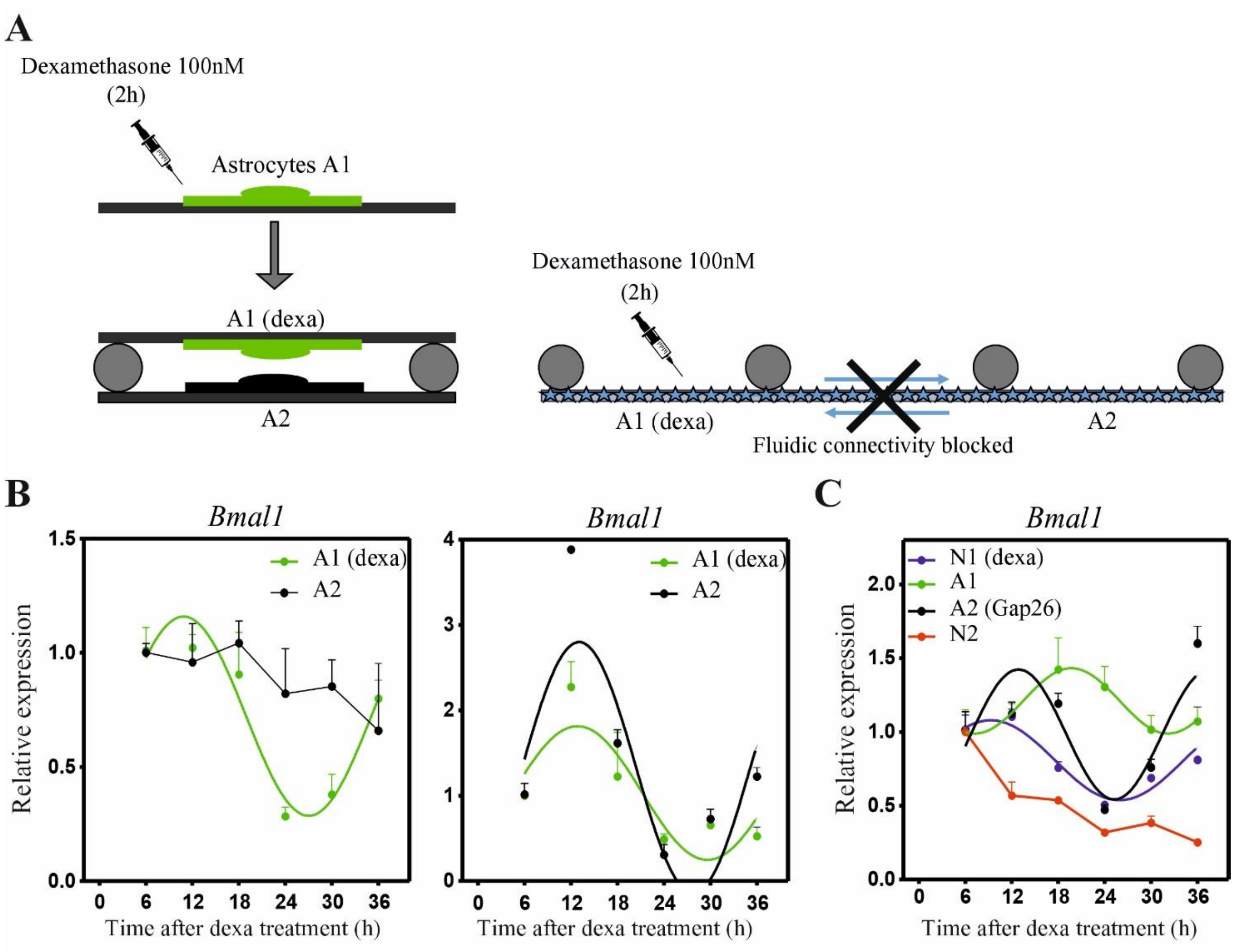

Supplementary Figure 4: Intercellular astrocyte communication for the astrocytes-mediated synchronization. A) Schematic representation of the experimental protocol. Astrocytes A1 and A2 not in contact (left) or in direct contact (right). B) *Bmal1* expression in A1 and A2 not in contact (left) or in direct contact (right). C) *Bmal1* expression when intercellular communication between astrocytes is blocked with Gap26 100 µM in A2. In B) and C), *Bmal1* was analyzed at the indicate time points by qPCR and the mean ± s.e.m. of the cosine-fitted curves from an experiment performed in triplicate is represented.

Supplementary Video: Compartmentalization testing by using a Coomassie Brilliant Blue dye. Top: case without vertical perfusion. As shown, the blue dye in the left well can flow in the right well. Bottom: case with vertical perfusion. As shown, the blue dye remains confined in the left well.
